# The impact of COVID-19 on access to harm reduction, substance use treatment and recovery services in Scotland: a qualitative study

**DOI:** 10.1186/s12889-022-12873-y

**Published:** 2022-03-15

**Authors:** Joe Schofield, Joshua Dumbrell, Catriona Matheson, Tessa Parkes, Angus Bancroft

**Affiliations:** 1 Salvation Army Centre for Addiction Services and Research, Faculty of Social Sciences, Colin Bell Building, University of Stirling, Stirling, FK9 4LA Scotland; 2 Faculty of Social Sciences, Colin Bell Building, University of Stirling, Stirling, FK9 4LA Scotland; 3 School of Social and Political Science, Chrystal Macmillan Building, 15a George Square, Edinburgh, EH8 9LD Scotland

**Keywords:** COVID-19, Substance use, Harm reduction, Opioid replacement treatment, Recovery

## Abstract

**Introduction:**

People who use drugs (PWUD) are considered vulnerable to COVID-19 exposure and the sequelae of infection due to their social circumstances, health conditions, drug purchasing, and substance use. They can depend on access to services that provide harm reduction, substance use treatment, recovery and support, and general healthcare. Social distancing measures and service restrictions posed significant challenges to the health and wellbeing of PWUD.

**Methods:**

Ethical approvals were secured. PWUD were recruited from voluntary sector homeless and housing, harm reduction, and recovery organisations across central Scotland. Data was collected via semi-structured interviews and analysed using the Framework Method.

**Results:**

Twenty nine PWUD participated and reported mixed experiences of the impacts of COVID-19 lockdown. Several benefitted from policy and practice developments designed to sustain or increase access to harm reduction services. Some PWUD reported improved access to substitute prescribing and/or appreciated being trusted to manage multiple take-home doses. Others noted the loss of regular in-person contact with treatment providers and dispensers. Access to recovery support was challenging for many, especially those unable to access or uncomfortable with online provision who experienced greater isolation. Lack of access to general healthcare services was common, and especially problematic for PWUD with chronic physical and mental health conditions.

**Conclusions:**

This qualitative research describes the impacts of COVID-19 social and service restrictions on PWUD in Scotland. These impacts were anticipated by policy makers and service providers. Effective and acceptable developments were shown to maintain and even increase service provision for PWUD. Developments were geographically dependent and significant challenges remained for many people. The learning generated can inform responses to increase service access and uptake in post-pandemic times.

## Highlights

- COVID-19 lockdown had significant impacts on people who use drugs (PWUD).
- Access to harm reduction, treatment and recovery services was negatively affected.
- Positive developments included rapid treatment access and continuity of care.
- Loss of therapeutic relationships with services and peers was a challenge.
- Nimble, person-centred responses were welcome, but unequally distributed

## Introduction

Novel coronavirus 2019 (COVID-19) is an infectious viral respiratory disease. Although anyone can become infected, the risks of experiencing the most serious disease outcomes are inequitably distributed among populations. A syndemic of COVID-19, chronic disease, and social determinants of health has been described in which the prevalence and severity of infection co-occurs, interacts with, and exacerbates, existing health and social conditions among already overburdened groups [1, 2].

People who use drugs (PWUD), including those who experience problematic use of opiates such as heroin, were considered potentially vulnerable to COVID-19 because of their social circumstances, comorbidities, and substance use. They are more likely to experience socioeconomic disadvantage, homelessness, and housing insecurity [3], imprisonment [4], and often face barriers to healthcare services including discrimination and stigma [5]. They are disproportionately affected by comorbidities that increase the risk of severe disease or mortality and can be immunocompromised because of untreated HIV or hepatitis C infection, liver disease, or due to substance use [1]. The social nature of drug use could also place them at increased risk of COVID-19 exposure. Purchasing and consuming drugs are often social activities that can involve visiting other peoples’ homes, using drugs in groups, and sharing substances and paraphernalia [6].

In response to the COVID-19 pandemic, the Scottish Government introduced a range of measures described as “lockdown” [7]. Lockdown placed stringent restrictions on the movement of individuals and the operation of services. Health and social care services were significantly affected due to staff illness, absence, and redeployment; direct pressures caused by COVID-19 disease; and the need to protect staff and clients from virus exposure [8, 9]. The COVID-19 pandemic emerged in the context of ongoing public health crises affecting PWUD in Scotland who were experiencing increasing drug-related deaths, the highest rates in Europe, and the need to increase uptake of harm reduction, treatment, and recovery services [10].

Policy makers and service providers recognised the potential for social distancing guidance to impact on services for PWUD. People were advised to collect sufficient injecting equipment provision (IEP) supplies to last 2 weeks and were provided with information on the cleaning and reuse of injecting equipment “as a last resort” [11]. The Crown Office and Procurator Fiscal Service (COPFS), which is responsible for prosecutions in Scotland, issued a statement of prosecution policy that extended the distribution of naloxone to non-drug treatment services [12, 13]. Services established home postal delivery of IEP and naloxone [14].

Substance use treatment provision, including prescribing from specialist services and dispensing at community pharmacies of opioid replacement therapies (ORT), was also affected. Early in the pandemic, Scottish treatment services were advised to prepare for potential disruption in medication supply [15]. Maintaining daily dispensing of ORT proved logistically challenging and presented risks for COVID-19 transmission in a range of settings. Policy and service responses included a shift to longer dispensing intervals, long-acting depot formulations, home delivery, and allowing friends or family members to collect ORT on behalf of individuals [15, 16].

This research explored the impacts of COVID-19 related changes on the availability and uptake of health and care services, particularly harm reduction, treatment, recovery, and general healthcare services, among PWUD in Scotland during the pandemic.

## Methods

A qualitative study involving semi-structured interviews with PWUD was conducted between May and November 2020. The research was designed to minimise the burden on services and their clients during this challenging time. Ethical approvals were granted by the University of Stirling’s General University Ethics Panel, The Salvation Army, and Turning Point Scotland.

Purposive sampling was used to cover a range of gender, age and treatment or active drug use status. Participants were recruited from a range of voluntary sector organisations that supported PWUD across four areas in central Scotland. The settings included a homelessness residential service (hostel/shelter), a stabilisation and housing service, a harm reduction service, and a peer-led recovery community. Participants were aged 18 or over and self-identified as currently using street drugs and/or receiving treatment for a substance use problem. Clients were recruited via service managers who were asked to discuss the project with eligible clients, seeking to include a balance of genders and people at different stages in relation to their drug use (currently using drugs and/or in treatment and recovery). Clients who wished to participate could either contact the research team directly by telephone or email or ask a member of service staff to pass on their details. No information was available on the number or characteristics of PWUD who were approached but declined to participate. Two experienced researchers (JD/TB) were responsible for liaising with and interviewing PWUD. Researcher JD had dual roles as a Community Researcher with lived experience of problematic drug use and homelessness and a specialist support worker within a homelessness service. He was known to participants at this service and was able to conduct in-person and telephone interviews in line with the risk assessment and national / University / service COVID-19 guidance. JD also conducted telephone interviews with participants from the other services. TB exclusively conducted telephone interviews.

Informed consent was obtained from participants before any data were collected. All participants were offered a £20 supermarket voucher to thank them for their contribution. The topic guide covered changes to drug use since the start of lockdown, access to/utilisation of drug-related and other healthcare services, and general impacts on physical and mental health. All interviews were audio recorded and transcribed in full. Interviews typically lasted around 30 min (range 11 to 48). Transcripts used pseudonymised participant codes and all names of people and services were replaced with pseudonyms.

Interview data were analysed using the Framework Method [17] in NVivo 12 [18]. An inductive approach to analysis was used in which a coding framework was iteratively developed using themes emerging from the data. JD initially read four transcripts in full and these were coded line-by-line to identify emerging themes which were then reviewed by TB. CM reviewed the coding framework. This framework was then applied to the full dataset. New categories were added if they emerged. Seven interrelated high-level themes were identified. Four of these concerned impacts on relevant health and social care services, which formed the basis for this paper: access to harm reduction, substance use treatment, recovery and support, and general healthcare services. Themes not reported here were not healthcare related and included impacts on drug markets, drug distribution networks and quality of drugs. The results and quotations presented here cover the range and depth of data collected. Data collection continued until data saturation was considered to be achieved by consensus of both interviewers.

## Results

A total of 29 interviews were conducted at which point data saturation was considered to be achieved as no new themes or variation in experience were being expressed. Everyone who consented to participate completed an interview and no one withdrew from the study. Table 1 provides a summary of interview and participant characteristics. Reported gender of interviewees is provided in quotations.

**Table 1:** Interview and participant characteristics

		*N* = 29
Interview type	In-person	10
	Telephone	19
Interview Location	Drop-in service	16
	Housing service	8
	Harm reduction service	3
	Recovery Community	2
Gender	Male	16
	Female	13
Age	20–29	1
	30–39	9
	40–49	15
	50–59	4

### Harm reduction services

Across the interviews there were descriptions of services adapting and targeting their harm reduction provision, often with a strong focus on people at risk of overdose. Developments included targeted outreach and home delivery of naloxone and IEP, enabled by the revision of legal and policy environments, including COPFS guidance supporting the supply of naloxone in a wider range of settings during the pandemic. Naloxone awareness was high among the sample. All participants had at least some knowledge of the product, including several trained to administer it. Roughly half of the interviewees said they had naloxone in their possession at the time of the interview and knew where to obtain additional supplies. Those who did not possess naloxone expressed an awareness of its utility and knew where to access a supply.

Many participants recognised the value of expanded access to naloxone, which could be supplied by a wider range of services during the pandemic and perceived this as a measure that would help keep them and their peers safe. Some expressed their appreciation.

*Yeah, they’ve still been open and available, they’re doing a good job, I’ve got to give hand up to that. (M, 46)*

*Aye, I mean, because like all the needle exchanges and things like that too, that’s one of the things that they do ask you, ‘have you got a naloxone?’ You know, have you done the training? No, well listen we could help you with that, and they offer places where you can go to get it done. (M, 38)*

Several participants reported that access to their usual IEP service continued as usual during COVID-19, albeit with social distancing restrictions. Messaging regarding pharmacy closures or reduced IEP provision was received and understood by several participants who used IEP services.

Participants also commented favourably on the upscaling of IEP sites and novel delivery methods during the pandemic. These included outreach and home delivery of equipment to people who were shielding and an expansion of the number and type of outlets.

*Yeah, well actually, there’s lots of posters up about it, yeah, yeah, there was lots of posters up in the chemist about it ( … ) Yeah, yeah, you just tell them and they can post it out to you. (F, 43)*

Despite initial concerns that pharmacy supplies might run out, participants generally reported only minimal disruptions to pharmacy IEP services and several pharmacies encouraged people to attend less frequently and collect sufficient supplies to meet their injecting needs.

*I was worried that the pharmacy would close right, but because I’m on a prescription ( … ) I always knew that (pharmacy) would always be open, but I was very uncertain that it would be just open for, for prescriptions, and I didn’t actually know that they’d be there to do like exchange, but through the pandemic ( … ) what they were actually telling you, if you went in twice a week, to get your, to get your exchange, what they would say is, double up on the stuff that you got, you know, if you were only basically taking yourself one day a week instead of two, that’s what they were telling you. (M, 42)*

Restrictions on non-essential travel, and the implications of having to justify being out on the street during lockdown, caused one participant to stop attending their preferred IEP pharmacy through fear of breaching the new laws.

*No, I moved exchanges. I go to the one closer to the house. (F, 37)*

Some participants reported benefitting from the development of a new, onsite IEP in their hostel, describing it as “*good*” and a place “*for [us] to be safe*”.

*[I] just get clean works from in here off somebody ( … ) even these in here are giving out clean works, or they have been doing. (M, 50)*

However, two participants preferred to access their regular IEP service due to the relative anonymity this affords.

*I prefer to use the pharmacy I’ve been using because I know, I know the way they work ( … ) I just try and keep myself away from this exchange, do you know what I mean? It’s because somebody clocks you doing needle exchange, and then they automatically think, oh, he’s, what’s he wanting needles for? He must have stuff ( … ) I can’t be doing with that. (M,42)*

One participant observed a high-risk group injecting situation and suggested this was due to restricted IEP pharmacy provision caused by COVID-19.

*I’ve seen it in a house, because they couldn’t get the chemist, because it was shut with the Covid, they couldn’t walk into the chemist to get needles, clean needles, and I seen people in a house, three fuckin people mate. One of they [those] people had HIV and they shared one needle between the three of them. (F, 44)*

Under lockdown, access to IEP was generally sustained, but arrangements and PWUDs’ experiences were location dependent.

### Substance use treatment services

COVID-19 developments in the sector included longer opening hours and more flexible service delivery e.g., support staff were able to liaise with designated General Practitioners (GPs) by text message and the GPs would provide outreach, seeing clients at the hostel or another service. Several participants spoke of the speed with which they, or people they knew, had been able to initiate ORT since the start of the pandemic, often taking days to have a prescription in place when previously it could have taken several weeks. In one city this was the result of service developments, planned pre-COVID and designed to reduce risk of drug-related deaths, whose introduction was accelerated due to the pandemic.

*Aye, there was a few of us that got rapid prescribed, the same day, or the following week, and one of my pals, it was the [Community Psychiatric Nurse: CPN] actually, went up where he was sitting begging, and she give him a drug test … and she went up and started him on a prescription that day … that stopped him begging and he’s in [shelter name], he’s doing alright now. (M, 44)*

Some treatment services switched patients from methadone to buprenorphine-containing products, including long-acting formulations and those containing naloxone, as a risk reduction strategy.

*I’ve begged and begged and begged that doctor for months for to try and get onto to subbies [Subutex], for to get off that methadone stuff … since this Covid started, and then boom, straight away, when I walked into [centre name], when I heard it was only for methadone, I gets a phone call right after it, and then it was on my script for subbies, what I’ve been trying to do for months and months and months. (F, 44)*

ORT dispensing arrangements also changed in response to the pandemic and several participants reported a shift from daily supervised consumption of their medication to being given multiple doses to take home. This shift was introduced to reduce interpersonal contact and potential COVID-19 transmission within Community Pharmacies. Some participants spoke favourably of this change, feeling more trusted to manage their own medication.

*I go every day, but I just take the bottle away with me, I get a wee measuring cup. I take half, I take half in the morning and take about, maybe about 7, 8 o’clock at night … It’s a bit of trust she’s gave me as well, you know, because I could just skelp the lot or save it up or whatever, but what’s the point in doing that. (M, 44)*

Others preferred the stability and structure provided by daily attendance and struggled with being given several days’ medication to take away and the temptation of having multiple doses at home.

*I was supervised, and then with the Covid thing, they started giving you it to take away … now that they’re trying to get us back in the chemist, I’m no doing that, because it’s still no safe, so I got to take away every day … I want to work towards picking up so many times a week … they were trying to put me down to twice a week, and I went no, 3 times a week, that’s right enough for me the now, because 2 times a week, it’s too much of a quantity for to be sitting there … I want to do it gradually, you know … I know what’s good for me. (M, 38)*

The dispensing of large quantities of ORT also posed challenges and some participants reported their peers were being approached outside the pharmacy and pressured to sell or give away their medication.

*A lot of them are getting threatened for their script around there … people come to the chemist to watch for you, so it’s pretty shit like. (F, 43)*

Some people reported tensions between substance use workers or prescribers and their patients. There were examples of whole groups of patients being penalised for the observed or suspected behaviours of others.

*The only thing that, that really I noticed changing was people getting it out maybe twice weekly or weekly or whatever, it’s just depending how, how the workers how much the workers would trust them, some people, some people have still to go daily, you know, but they were few and far between I think that was for people that maybe was going into them, telling them a load of lies or whatever. (M, 49)*

Face-to-face contact with some workers in the community was disrupted and several participants reported cessation of contact with their healthcare team.

*Before the pandemic, [I saw my CPN] once a month, and I haven’t seen her since all this happened. (F, 33)*

Consultations, where they took place, were generally conducted by telephone or online video calls. Several participants expressed their understanding of the significant pressures affecting NHS staff during the pandemic.

*I’ve barely seen [CPN], or spoke to her, or whatever, because I imagine she’s been run off her feet, I mean every time I was with her, she was having to get up, she was getting another call for somewhere else, and these calls were rapid, eh so, the lassie was run off her feet, so we barely got to see them. (F, 44)*

One person described their sense of isolation, disengagement from recovery, and an extended period of unplanned withdrawal that resulted from them not knowing how to contact their care provider. They were only able to alert their treatment service and reinstate their treatment through a family contact.

*I stopped, I tried to stop during lockdown I tried to stop taking heroin, diazepam, I was taking pregabalin at the time as well actually, and I was on methadone, and I just, because I wanted to stop, I stopped them all, I stopped going for my methadone, and didn’t know how to get back, I had drugs in the house, I had drugs there, but I was, I’m stubborn, and I was determined that I wasn’t taking them, and I was ill for like 2 weeks, and because I didn’t know how to contact anybody, because of lockdown, everywhere was shut, didn’t know how to get in touch with my care manager … my cousin works in recovery … she knows my care manager, and she spoke to him and … he had a script in the chemist that day for me. (M, 32)*

### Recovery support services

A third of participants reported accessing non-statutory service group activities e.g. support groups delivered online using virtual platforms. One interviewee reflected on the significance of these groups, referring to them as “*a life saver for a lot of us*”. The issue of digital exclusion was identified as a key factor that determined whether participants and their peers were able to access peer-led community support.

*I found the online stuff okay, and that, I was okay that way, but for others I know it would be harder, especially the ones that weren’t in the recovery community, or just about to start, it’s, it would be more difficult for them to just go into a meeting what they had no idea about. (F, 47)*

*It’s just a nightmare, I hate it, I hate technology, I always, I hated it from day one. (M, 43)*

One interviewee spoke favourably of the welcome he received from staff at an online peer support group, and the accessibility of this form of support.

*One of the admin people that phoned me, phoned me that day that I joined and what have you, told me how it works and things like that, so aye, they made me really welcome, sort of thing, so I, that’s been a huge part of help as well, that being there every day from Monday to Sunday sort of thing. (M, 49)*

Others appreciated the flexible response demonstrated by the development of online options to engage in recovery communities and activities, including the ability to access international online meetings at times when Scottish groups and services were not operating.

*Even the first Zoom meeting I went to a couple of weeks ago, I was like, you know, I’ll go, but I’ll no talk and then I went and when I spoke, I, see the way I felt after the meeting, like a weight had been lifted off my shoulders, it was, it was crazy, and again, I mean no drug in the world can give you that feeling. (M, 32)*

*You can go into a Zoom meeting 24/7 basically ( … ) if you feel a wee bit low or you feel a wee bit maybe, an urge or a temptation or anything like that, there’s always a Zoom meeting there, and it’s open and you’re always welcome in, so I’ve found that quite good as well, I found myself in meetings in Boston and things like that, you know. (M, 49)*

This included examples of voluntary sector services providing digital access to people who would otherwise be excluded, including one person who received a data plan and access to digital entertainment to help them and their family during the pandemic.

*I had no Wi-Fi for a while, so even the [third-sector organisation] even gave me some data, so that my little boy had some data on his tablet, and I was able to connect into that to get on WhatsApp groups ( … ) they gave me a Now TV box, so the wee man [her son] had cartoons to watch, and they bought you blenders and you know, fruit to make smoothies and that after fitness, they done a lot. (F, 43)*

Conversely, several people reported experiences of loss and isolation when recovery group meetings ceased or became inaccessible due to moving online during the pandemic. Several of our participants lived alone or in hostel accommodation which was likely to have been isolating even before pandemic conditions.

*Yeah, well I was doing [mutual aid group] before the Covid and then, and that’s something else that I miss too. (M, 58)*

Most interviewees reported being negatively impacted by the lockdown measures, particularly through the loss of, or reduced access to, social supports. Three participants who were active within their respective recovery communities pre-lockdown spoke of relapsing into illicit drug use due to significant reductions in community-based support and recovery-focused activities. One individual linked her relapse into using heroin directly to the physical closure of the services central to her recovery.

*Well I was actually off it [heroin], at the time, but it was only when you had to be stuck in the house that I started it again. (F, 43)*

### General healthcare services

Almost all participants reported experiencing a range of health problems during the pandemic, including long-term conditions associated with poverty and substance use, and many with multiple coexisting problems. These included chronic pain, diabetes, weight loss and skin problems due to poor diet, hepatitis C infection and treatment side effects, respiratory and cardiovascular problems including breathing difficulties and chronic obstructive pulmonary disorder.

Several people reported problems caused by reduced access to healthcare services in community and hospital settings.

*I got a phone call this morning from going yesterday and I’ve to go at 2 o’clock today, to see a doctor and that’s been a week, I’ve been trying to get a doctor. (M, 46)*

*Well I’ve no been back for my scan to the hospital, my breathing’s knackered, I know that there’s problems with my heart, I’ve not got, they’ve no had me back to the hospital for that yet, well obviously my kidney, my kidney’s been knackered for ages, that worries me because my ma was on dialysis eh, for nearly a year … I have been worried sick about it, and I’ve no been able to see a doctor about it. (F, 44)*

A key concern for several participants was access to dental care, particularly for some who had long-term problems. One participant attributed their increased drug use to coping with tooth pain.

*I suffered from bad toothache, which again, when I suffered from toothache, I only knew one way to get, to get rid of the toothache and that was use … I used a lot, and then I eventually phoned the dentist, and I got put, I got emergency appointments, but they were only taking teeth out. (M, 32)*

Several participants appreciated healthcare providers who responded to lockdown by introducing telephone or online video consultations.

*I’ve had great, I’ve been on phone consultations when I needed them, when lockdown was on, but last week, or the week before, I think I got, I managed to get a consultation actually with the doctor now from, and it was the first consultation with my doctor, but I never worry, because every time I rang, or even if I couldn’t get him, we’d leave an email for him, and [he] would get straight back to us, because he knew my situation, so it was never a problem like no. (M, 50)*

Others, however, felt difficulty in speaking on the phone and felt that it was detrimental to their mental health.

*I was using [service name], and they would have to get me over the phone, or at, sometimes we be a video call … social work as well, they have to get me over the phone, so that kind of was a bit tricky … I’ve got a lot of anxiety, that kind of, it made it kind of worse like, like I don’t really like talking over the phone or anything like that, so it was kind of, aye. (F, 43)*

## Discussion

This study explored the impacts of COVID-19 on PWUD in Scotland regarding the availability and quality of harm reduction, treatment, recovery, and general healthcare services. Despite contingency planning and policy responses, the early phase of the pandemic in Scotland was characterised by disruption to services and increased isolation for people experiencing homelessness and substance related problems [3, 19]. These mirrored experiences across the world, [1, 20, 21] and are supported by emerging quantitative research in Scotland [22]. Participants described challenges they experienced accessing services at a time of personal and social disruption and uncertainty. Efforts to sustain and increase access to treatment and harm reduction services were noted, although experiences varied depending on geographical location, and provider-led solutions such as increased take-away doses of ORT sometimes had unintended consequences for PWUD. Access to social supports and general health and care services was suboptimal, especially for those unable to access online provision. In line with work elsewhere, the results suggest that the COVID-19 pandemic and drug-related harms entangled to form overlapping public health emergencies [23, 24]. A dominant theme from our results is that social responses to COVID-19 produced conditions that exacerbated PWUD’s proximity to risk and diminished their capacities to respond, especially through the fracturing of social and service-based therapeutic relationships and supports [8, 25]. In some cases there were additional pressures from, and impacts on, their capacity to care for dependents.

In recent years, Scottish policy developments have sought to increase the accessibility, quality and uptake of public health-informed services for PWUD including guidelines for IEP services, a national naloxone programme, and recovery-oriented systems of treatment and care [26–28]. Macro-environmental responses to COVID-19 prioritised social distancing and isolation measures designed to prevent and control infection [29]. Our participants described how these disrupted the availability and accessibility of health and care services for PWUD, illustrating how national public health policies exacerbated risks for individuals and communities. Restricted access to IEP led to the reuse and sharing of injecting equipment which is associated with bacterial and blood-borne virus infections. Prescribing changes, designed to reduce the frequency of attendance at community pharmacies and other dispensing services, resulted in people being given multiple days medication to take home, increasing the risk of diversion and overdose. Of concern, some participants reported being exposed to social risks that included being coerced into giving away medication when leaving the pharmacy with a supply of ORT. Anecdotal reports compiled by one Scottish Health Board noted issues including: methadone “leaking” on to the illicit market; people not taking ORT as prescribed; people sharing prescriptions with others; and intravenous preparation of an ORT wafer designed to dissolve on the tongue [30].

At the macro-level policymakers, European and Scottish organisations recognised the need to ensure continuity of care and to ameliorate the risks of harms that could result from reduced access to services for PWUD. Health and care commissioners and providers were advised to consider the impact that the withdrawal or interruption of service provision could have on this vulnerable section of society and to respond nimbly and flexibly where possible [6, 13, 15, 16]. The Scottish Government provided over £2 million for long-acting depot buprenorphine injections and residential rehabilitation for people leaving prison, and the Lord Advocate issued a statement that relaxed the rules regarding who could supply naloxone [13]. These high-level changes created opportunities for personal benefits and reductions in harm that PWUD experienced at personal and social levels. The move towards rapid, and in some cases same-day, prescribing was noted, especially in comparison to the extended process of entering treatment before the pandemic. Some PWUD appreciated not being required to attend for daily dispensing and being trusted to manage their own medication schedule, enhancing their sense of agency and active management of their treatment, which has been shown to improve adherence [31].

Before the pandemic, loneliness and social isolation were recognised as challenges for PWUD and known to be associated with poor mental health and other adverse health outcomes [32]. In line with other research, the withdrawal of face-to-face contact with service providers and peers was noted by many people we interviewed, exposing them to additional harms through interrupted treatment and loss of contact with formal and informal psychosocial supports [33–35]. Policy and service-level responses sought to ensure continuity of care through a shift to telephone and online video calls. Whilst several participants welcomed this development, others were uncomfortable or excluded from this virtual contact.

### Strengths and limitations of the study

This study reported the experiences and views of PWUD from several areas in Scotland during the first 7 months of the lockdown. Participants included a balance of males and females aged between 28 to 56, people who were currently using drugs and/or in treatment and recovery. The range of participant viewpoints provided a rich picture of the impacts of COVID-19 for a cross section of PWUD. Recruiting current service users may have skewed the sample towards people who maintained service engagement in the pandemic, however most participants were recruited from housing services, so were not skewed towards those actively engaged in harm reduction, treatment, or recovery services.

Several interviews were conducted by JD, who had dual roles as both a community/peer researcher with lived experience of problem drug use and a specialist support worker at the hostel in Edinburgh. JD’s role included the provision of harm reduction information and support. Once the data collection had concluded, participants were provided with support and advice to help them to address urgent problems they had disclosed during the interview. We are confident that this additional support did not contaminate the data collected and was a highly important contribution to reducing acute risks for our participants.

### Recommendations

Policy makers, service planners and providers should review the experiences of PWUD under COVID-19 to understand and address the harms caused to some. Learning from the experience of rapid access to ORT, remote clinical care and peer-led recovery support could be applied to reduce barriers to care post-COVID, but issues of digital exclusion and personal preference should be considered.

## Conclusion

This study aimed to understand the impacts of COVID-19 on PWUD in Scotland. Many people experienced significant challenges resulting from the loss of access to key harm reduction, treatment, recovery and general health and care services and therapeutic relationships. In some cases, this loss of support led to relapse into substance use and exacerbation of pre-existing physical and mental health problems. Early in the pandemic, policy makers, service planners and providers noted the importance of ensuring continuity of access where possible, and several examples of contingency planning and innovation in delivery were apparent. Clients appreciated the rapid introduction of improved access to ORT and the expansion of IEP and naloxone into new settings, although such developments were not equitably distributed across the areas included in our study. Providers attempts to ensure continuity of care through telephone and online consultations were welcomed by some clients, but digital exclusion and discomfort with impersonal channels were a barrier for others. Several participants reported a complete loss of ways to contact their care providers. The inaccessibility of general health and care provision, especially dental care, was a common challenge and reduced quality of life for many.

## Data Availability

The data that support the findings of this study are available on request from the corresponding author JD. The data are not publicly available due to them containing information that could compromise research participant privacy/consent.

## Abbreviations

COVID-19: coronavirus disease 2019
PWUD: People who use drugs
IEP: Injecting Equipment Provision / Provider
CPN: Community Psychiatric Nurse
COPFS: Crown Office and Procurator Fiscal Service
ORT: Opioid Replacement Therapies
GPs: General Practitioners
